# A prospective cohort study measuring cost-benefit analysis of the Otago Exercise Programme in community dwelling adults with rheumatoid arthritis

**DOI:** 10.1186/s12913-018-3383-4

**Published:** 2018-07-20

**Authors:** Siyar Abdulrazaq, Jackie Oldham, Dawn A. Skelton, Terence O’Neill, Luke Munford, Brenda Gannon, Mark Pilling, Chris Todd, Emma K. Stanmore

**Affiliations:** 1 E K Stanmore School of Nursing, Midwifery and Social Work, MAHSC (Manchester Academic Health Science Centre), University of Manchester, Jean McFarlane Building, University Place, Manchester, M13 9PL UK; 2 Arthritis Research UK Centre for Epidemiology & NIHR Manchester Musculoskeletal Biomedical Research Unit, Manchester Academic Health Science Centre, The University of Manchester, Manchester, M13 9PL UK; 3 School of Health and Life Sciences, Glasgow Caledonian University, Cowcaddens Rd, Glasgow, G4 0BA UK; 4 Centre for Health Economics, Institute of Population Health, University of Manchester, Manchester, M13 9PL UK

**Keywords:** Rheumatoid arthritis, Health economics, Falls, Injury, Costs, Falls prevention, OTAGO, Prospective

## Abstract

**Background:**

Falls are one of the major health problems in adults with Rheumatoid Arthritis (RA). Interventions, such as the Otago Exercise Programme (OEP), can reduce falls in community dwelling adults by up to 35%. The cost-benefits of such a programme in adults with RA have not been studied.

The aims of this study were to determine the healthcare cost of falls in adults with RA, and estimate whether it may be cost efficient to roll out the OEP to improve function and prevent falls in adults living with RA.

**Methods:**

Patients with Rheumatoid Arthritis aged ≥18 years were recruited from four rheumatology clinics across the Northwest of England. Participants were followed up for 1 year with monthly fall calendars, telephone calls and self-report questionnaires. Estimated medical cost of a fall-related injury incurred per-person were calculated and compared with OEP implementation costs to establish potential economic benefits.

**Results:**

Five hundred thirty-five patients were recruited and 598 falls were reported by 195 patients. Cumulative medical costs resulting from all injury leading to hospital services is £374,354 (US$540,485). Average estimated cost per fall is £1120 (US$1617). Estimated cost of implementing the OEP for 535 people is £116,479 (US$168,504) or £217.72 (US$314.34) per-person. Based on effectiveness of the OEP it can be estimated that out of the 598 falls, 209 falls would be prevented. This suggests that £234,583 (US$338,116) savings could be made, a net benefit of £118,104 (US$170,623).

**Conclusions:**

Implementation of the OEP programme for patients with RA has potentially significant economic benefits and should be considered for patients with the condition.

## Key messages

1. This is the first study that gives detailed analysis of healthcare cost of falls in adults with RA and estimates potential cost-savings.
2. Cumulative medical costs for 598 falls was £374,354 (US$540,485), average estimated cost-per-fall £1120 (US$1617).
3. The findings strengthen the case for the delivery of an evidence-based falls prevention programme for adults with RA.

## Background

Rheumatoid arthritis (RA) is an inflammatory disease of unknown cause that first targets synovial tissues, cartilage and bone. It is the most common form of immune-mediated arthritis affecting approximately 1% of the adult UK population [1, 2], with a global prevalence of 0.24% [3]. Symptomatic patients with RA present with joint pain, swelling, muscle weakening with fatigue and reduced functioning [4–8]. In the community, falling is a problem especially among adults aged 65 years or older, for whom falls are the main cause of both fatal and non-fatal injuries [9]. It is estimated that 30–35% of people in the community aged 65 and above have at least one fall per year [10, 11]. In adults with RA the risk of falling is even greater, with the annual incidence rate estimated to be between 10 and 54% [4–7, 12–18] and in contrast to those without RA the risk appears to be broadly similar across the age bands [19].

Most of the injuries resulting from a fall are non-fatal (e.g. bumps and bruises), but approximately 10–25% of falls result in more serious injuries such as hip fractures, head trauma or internal bleeding [9, 20]. Falls can affect a person’s morbidity and quality of life and also impact the health care system in terms of medical costs [9, 21, 22]. Falls are a common cause of Emergency Department visits, acute care admissions and hospitalisation among adults aged 65 years and over [22–24]. Apart from the acute care costs to consider there are also the social care costs which, according to estimates from the UK Department of Health’s economic evaluation, will incur ongoing costs of £1872(US$2702) per fracture patient, per year [25].

Many of the risk factors for falls, such as poor balance and gait or mobility impairments, can be improved by exercise [9]. Implementing effective prevention strategies could therefore potentially reduce the risk of falling, decrease the incidence of falls and reduce associated health care costs [26]. There is abundant evidence that exercise programmes that improve balance muscle strength and walking ability are effective in preventing falls [27–30]. Clinical trials provide evidence that an exercise programme as a single intervention can prevent falls in older adults living in the community [30–32].

The Otago Exercise Programme (OEP) is considered for implementation in patients with RA because it has demonstrated to be one of the most beneficial programmes to prevent falls [30, 33]. The programme consists of individually tailored muscle strengthening and balance retraining exercises with increasing difficulty combined with a gait-improving programme. The aims of the programme are to improve patient’s strength and balance and increase their confidence in carrying out everyday activities without falling. The programme has the greatest impact among high-risk groups; such as those with a previous fall and those aged 80 and above [31]. In the four trials studied with 1016 people ages 65 years to 95 years in nine cities and towns in New Zealand, the OEP reduced the rate of both falls and fall related injuries by 35% [30, 33]. A more recent systemic review and meta-analysis (88 trials with 19,478 participants) showed similar strong evidence that exercise that challenged balance and involved more than 3 h/week of exercise led to a 39% reduction in falls [32].

Trained physiotherapists or nurses are able to deliver the programme in the home setting. Patients are shown how to do a set of in-home exercises tailored to their needs during a one-hour visit and 3 to 4 half hour visits over the first 2 months. The exercises take approximately 30 min to complete. They are encouraged to walk outside twice a week and to complete the exercises three times a week. The aim of implementing this programme is to improve health and wellbeing of people by preventing falls and fall related injuries and reducing the impact on the healthcare services. The proposed net financial benefit would be that the averted healthcare costs outweigh the cost of implementing the programme. Such financial information would be beneficial in determining whether investing in the OEP as an intervention to prevent falls would provide a positive return of investment (PRI) for the National Health Service (NHS) or other such health providers.

To date there are no studies which have looked at interventions to reduce the risk of falls in adults with RA. Assuming similar benefits of the OEP programme as those without RA we looked at the potential cost savings if such a program were implemented. We used prospective follow up data on falls and determined the costs associated with falls in men and women with RA.

## Methods

### Study design

This study reports the follow up results from a prospective cohort study that was designed to determine the incidence and risk factors for falls in adults with RA (Stanmore et al., 2013). The participants in this study were patients who were referred from four rheumatology clinics in the North West of England during the years 2008 and 2009. Participants were followed up for one year with monthly falls calendars, telephone calls and self-report questionnaires on falls that included questions on the injuries incurred [34]. The baseline measurement was completed by *n* = 559 and *n* = 535 completed the 1-year follow-up. The timeline of data collection was between the years 2008–2010, further information about methods and participant demographics can be found in Stanmore et al. 2013 [7].

### Participant inclusion criteria

Participants were included if they had a diagnosis of RA (based on the 2010 American College of Rheumatology classification criteria for RA). All participants were over the age of 18, with the ability to give informed consent.

### Measurement of fall and injuries

All participants were given preaddressed, prepaid daily falls calendars which they posted monthly. Participants who reported a fall (or if they needed prompting to return the falls calendars) were telephoned to gain further information about the fall. A standardised questionnaire was completed by trained research nurses at the telephone interview to record details of the fall [34]. Falls were defined as, ‘an unexpected event in which participants come to rest on the ground, floor or other lower level’ as per the Prevention of Falls Network Europe (ProFaNE) which ensures that trips or stumbles are excluded [35]. The questionnaire included questions about factors including type of fall, type of injury, severity of fall, call out for an ambulance, requirement to attend A&E services or a stay overnight in public or private hospital. Other questions included whether their fall resulted in permanently moving to a care home or whether they had seen a doctor or other health professional. The standardised questionnaire also requested information regarding specific injuries (head injury, dislocation of a joint, fracture of a bone, stitches required, and presence of internal bleeding) or any other resources used as result of fall.

### Classification of falls

In order to estimate the cost of one fall, the seriousness of that fall and the services that were used in each fall episode had to be determined. Falls that were reported were verified by telephone calls and followed up to gather more information. This was used to classify the fall according to the severity of the injury, of which there were three options: no injury, moderate injury, and severe injury. If the severity of the fall was reported as serious or if the fall resulted in a fracture; a head injury with admissions to hospital or if stitches were required the severity of the injury was categorised as serious. The injury was moderate if the severity of the fall was reported as moderate and medical help was sought from outpatient clinics or if there was a head injury with bruising or sprains.

### Economic evaluation

#### Estimating cost of the fall related injury

The perspective of the economic analysis is that of the English NHS. To estimate the direct health care costs resulting from fall injuries, the National Schedule of Reference (NSR) cost provided by the NHS organisations from the financial year 2013/2014 was used [36].The cost for each injury (i.e. head injury or hip, wrist, knee, hand, lower arm fractures) and the services provided by the hospitals (hospitalisation, ambulance use, A&E attendance) were considered. As per National Institute for Health and Clinical Excellence (NICE) additional costs for x-rays and CT scans were added where the head injury or fracture was moderate or serious and required A&E attendance or hospitalisation [37] .The cost of inpatients admission was multiplied by the number of nights spend in hospital. One night on the ward in a public hospital included radiology, laboratory blood services, pharmacy products, hospital social workers, and physiotherapy and occupational therapy costs. The NSR included overhead costs (catering, cleaning, heating, telephone, lighting, laundry, administration, orderlies, and computing).

#### Estimating cost for the Otago Exercise Programme

The cost for implementing the OEP was estimated using 2015 financial records of Health and Social care from the Personal Social Services Research Unit (PSSRU) [38]. These are national estimates of staff costs in the NHS and include: the cost of wages and salaries. Additional costs included equipment (ankle cuff weights, instruction manual for trainers), on-going training and quality control courses for the physiotherapist, intervention costs (labour and travel time), telephone calls, and overhead costs. The costs are inclusive of government goods and services tax, and they are reported in British Pounds and US dollars using March 2016 converting rates. The costs for recruiting the exercise instructors were not included because the assumption was made that existing staff in the NHS can deliver the exercise programme. There was also no value put on the time patients spend exercising using the given intervention as it was assumed that the activities were done in their leisure time. The estimated overhead costs used was 19.31% of expected resource use, this percentage was used as it is the average reported for all hospitals and health services [38]. This additional cost is supposed to represents the support services used by the NHS for it to run effectively and includes administration and human resources. It is important to note that integrated care was not a feature in this study.

#### Analysis

For each fall the number of injuries is multiplied by the health care cost of that particular injury. The total cost of all 598 falls is obtained by adding all individual injury costs. Alongside this, an estimation of the cost is made for implementing the OEP. Previous studies measuring the effectiveness of the OEP has shown a 35% reduction in the number of falls and fall related injuries in the OEP group compared to the control group [31]. Therefore this would suggest 209 falls would be prevented. In the analysis the percentage difference is calculated between the total health care cost of 209 falls and the cost of implementing the OEP for all 535 participants. The resulting percentage difference indicates the potential savings from implementing the OEP.

## Results

### Participant characteristics

Full details of the participant demographics and characteristics have been described elsewhere (Stanmore et al., 2013). In brief, 69% of the 559 participants were women (*n* = 386) and the mean age of the participants was 62 years (SD = 13.6). The majority of participants were married or living with a partner (*n* = 378, 70%), were born in the UK and of white British ethnicity (*n* = 544, 97%). More than half of the participants were retired (*n* = 327, 60%), 15% were unable to work due to their disabilities (*n* = 82) and only 24% of the participants continued to be employed (*n* = 134).

### Falls

**Fig. 1:**
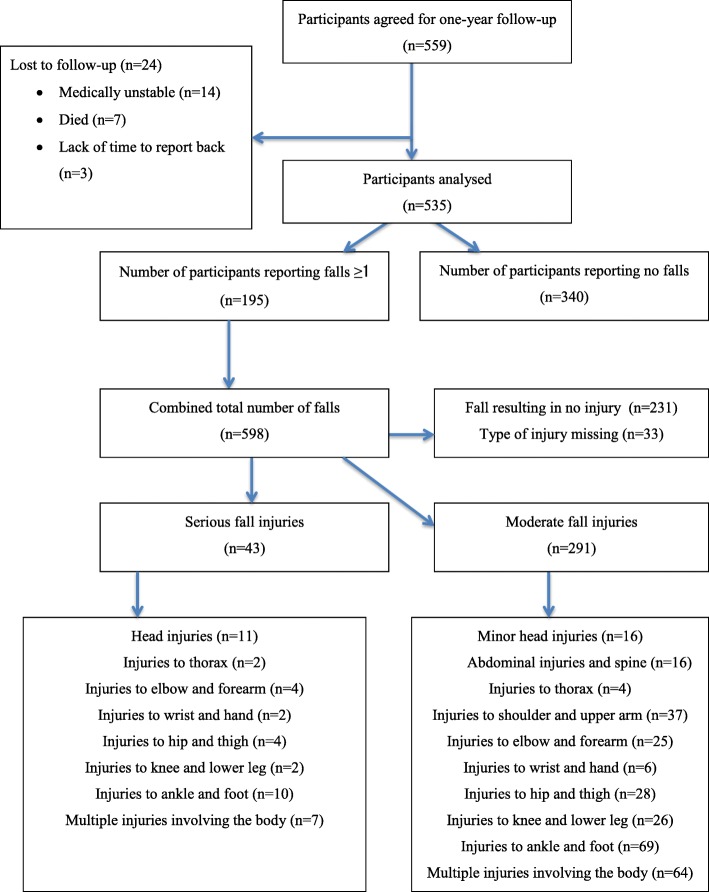
Flowchart diagram showing type of injuries

After 1 year follow-up 195 of the 535 participants reported at least 1 fall. In total there were 598 self-reported cases of falls with an average of 1 fall per participant, 43 (7.2%) reported as being serious, 291 (48.8%) as moderate, and 231 (44%) of falls resulted in no injury and in 33 the type of injury was not reported. Amongst the fallers the average number of falls was 6 falls, with a range of 1–40 falls. A flowchart of participants with type of injuries is shown in Fig. 1.

### Healthcare cost of falls

**Fig. 2:**
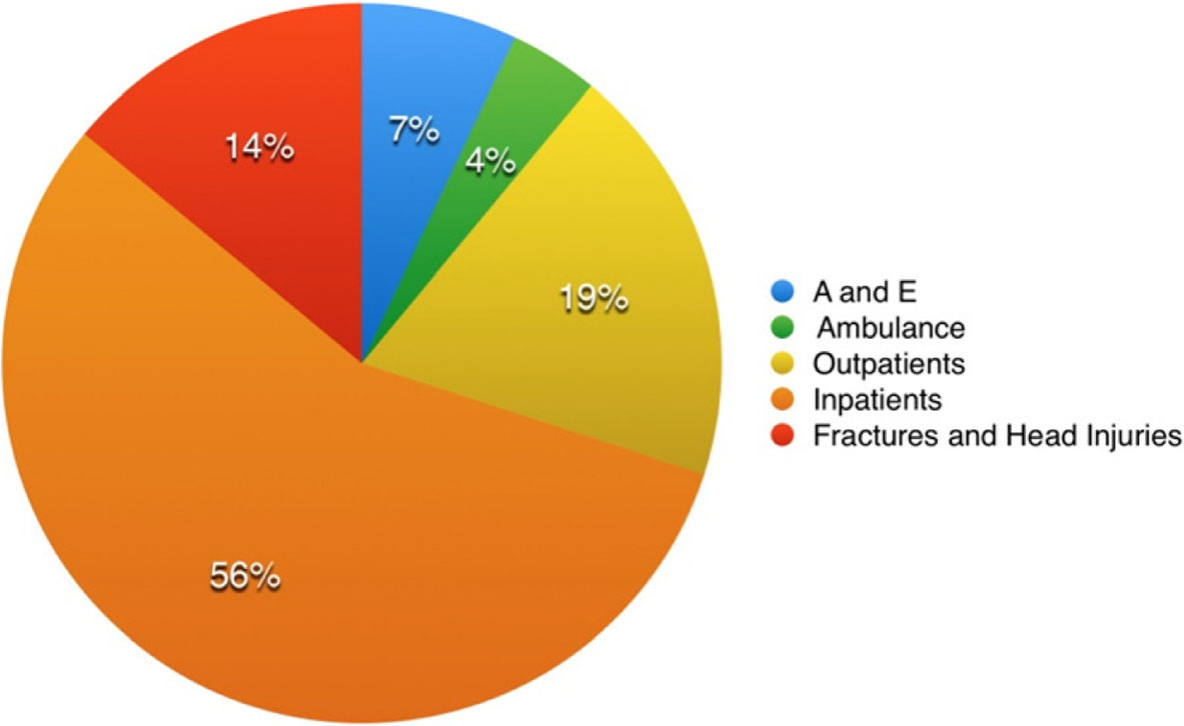
Chart showing spread of cost in health care costs

The direct medical cost to the National Health Service (NHS) of the 56%(334 cases) of falls that resulted in the use of health services was estimated to be £374,354 (US$540,485) or £1120 (US$1617) per fall. A detailed breakdown of costs of falls information is provided in Table 1. Studies conducted in New Zealand have shown that the cost per fall can range from £1214 (US$1752) to £2023 (US$2913) using 2016 conversion rates. A spread of costs spend on health service usage is shown in Fig. 2.

**Table 1:** The costs of various health care services utilized as a result of a fall

Action	Cost per individuals	Number of Patients using services	Total used services (in GBP)
Ambulance	230	17	3910
Visit to A and E	736	33	24,288
Number of nights in Public Hospital	698	259	180,782
Number of nights in a private hospital or rest home	75	4	300
Visit to doctor	111	86	9546
Stitches	468	6	2808
Injury with Haemarthrosis (Bleeding into join space)	2690	2	5380
Head serious injury	869	11	9559
Head moderate injury	608	16	9728
Fractured Ribs serious	11,347	2	22,694
Fractured Back serious	16,820	2	33,640
Fractured Lower arm	2511	1	2511
Fractured wrist	1825	3	547
Fractured hand	1906	3	5718
Fractured hip	13,408	3	40,224
Fractured knee	5770	2	11,540
Fractured ankle	2621	1	2621
Fractured toe	1118	4	4472
Estimated Radiography cost	93	17	1632
Fracture Knee Rehabilitation	556	2	1112
Fracture back rehabilitation	493	2	986
Rest home rehabilitation	356	1	356
			Total Cost: 374354.00

### Cost of OEP

Table 2 shows the values for the costs items for implementing the OEP.

**Table 2:** Table showing cost units of items in the Otago Exercise Programme

Activity	Resource Type	Type & Units	Cost/Unit	Annual Cost	Cost Per Participant per year (*N* = 535)
Equipment	Materials	2 x Ankle Cuffs Weights 535	Average £17.40 ($25.12)	£9309.00 ($13,439.86)	£8.70 ($12.56)
Training course for 27 PTs	1 Lead PT	Instruction 27 h	£34/h ($49/h)	£918.00 ($1325.36)	£1.72 ($2.48)
	Materials	2 Instruction Manual for LPT	£40.00 ($57.75)	£80.00 ($115.50)	£0.15 (£0.22)
		Ankle Cuff Weights			
Intervention	PT Labour	3 h per participant per session	£34/h ($49/h)	£54,570 ($78,785.43)	£102 ($147.26)
	PT Travel Time	1 h per participant per session	£34/h ($49/h)	£18,190 ($26,261.80)	£34 ($49)
	LPT Quality control check	27 LPT QCC	£34/h ($49/h)	£918 ($1325.36)	£1.72 ($2.48)
	PT Telephone Calls	0.75 h per participant per session	£25.50/h ($36.82)	£13,642.50 ($19,696.36)	£25.50 ($36.82)
TOTALs				£97,627.50 ($140,949.70)	£182.48 ($263.46)
Overhead Costs			19.31% of resources use	£18,851.87 ($27,217.40)	£30.25 ($43.67)
Total after overhead costs				£116,479.37 ($168,167.10)	£217.72 ($314.33)

PT-Physiotherapist

LPT- Lead Physiotherapist

Assumptions were made for the exercise programme:

- Current NHS Physiotherapists to implement the OEP.
- The lead physiotherapist would train a physiotherapist in one hour
- 27 physiotherapists would be trained in one year.
- Each trainee physiotherapist would have one-hour quality control check with a lead physiotherapist.
- The number of lead physiotherapists can vary but for ease it is kept as one here.

**Fig. 3:**
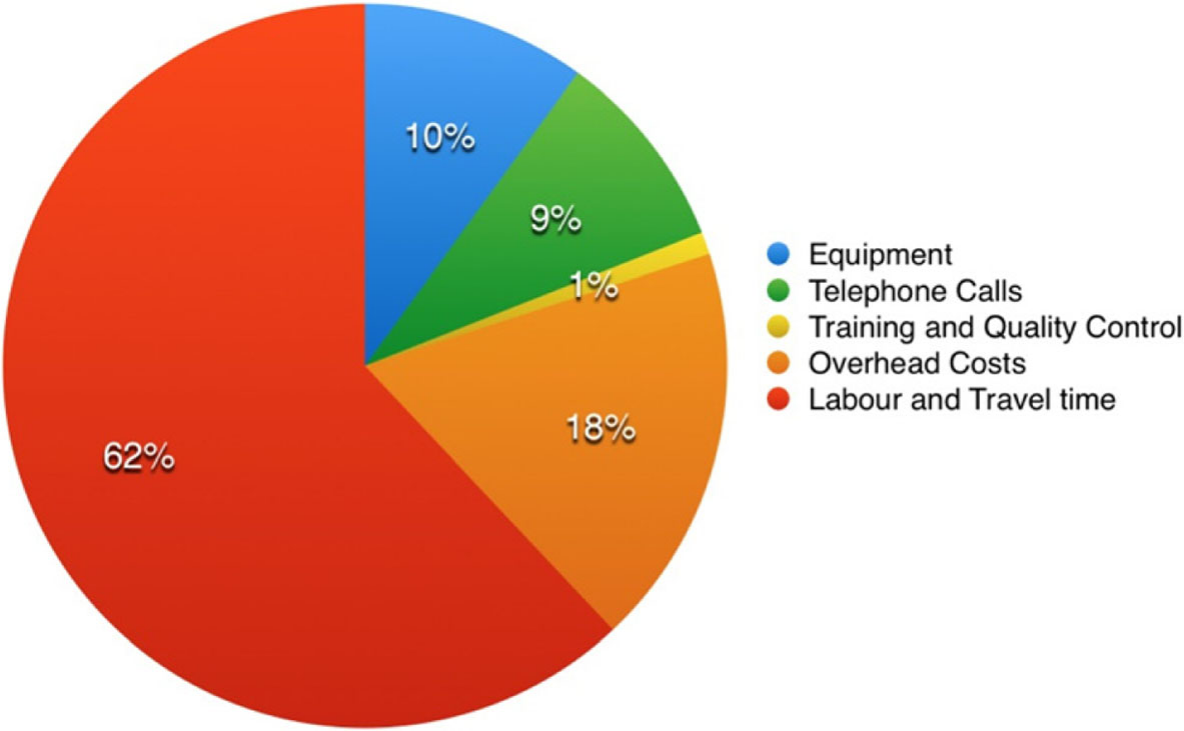
Chart showing spread of cost in the Otago Exercise Programme

Under these assumptions, the programme cost £116,479 (US$168,504) or £217.72 (US$314.34) per person to deliver to 535 participants for 1 year. Figure 3 shows the spread of cost for implementing the programme.

### Cost-benefit analysis

The average expected benefit would be £903 (US$1304) per participant. Previous studies measuring the effectiveness of the OEP has shown 35% reduction in the number of falls and fall related injuries [33]. In terms of healthcare cost analysis this would mean that out of the 598 falls 209 falls could be prevented. If 209 fall are prevented where each fall cost £1120 (US$1617) a saving of £234,583 (US$338,116) is made, and a return investment of £118,104 (US$170,623). The implementation of the programme estimated in the UK would bring more than a 100% return of investment (ROI), thus for every £1 (US$1.44) spend in healthcare 1.01 (US$1.46) pound would be returned. This ROI would be obtained from a reduction in ambulance use, ED attendance, hospitalisation and outpatient costs.

## Discussion

This study shows a high economic benefit of the OEP when delivered to community dwelling adults aged 18 years and older; it estimated a yielded ROI of more than 100%. The yielded return is obtained by comparison with the healthcare costs of £1120 (US$1617) per fall, for which the costs was obtained from the financial year 2013/2014. This value is based on the assumption that after an injury the individual used certain health services, for instance if they had a fractured hip it is assumed that they received hip surgery.

There is no literature on the direct cost of falls in patients with RA. In this study the estimated average healthcare cost per fall in patient with RA is £1120 (US$1617). In countries such as Finland and Australia the average healthcare cost per fall for people 65 and above is between £724–£2492 (US$1049–$3611), and this is regardless to whether the fall required hospitalisation [24].

In our study the OEP cost £217.72 or $314.34 (US Dollars) per person. Other studies in the US have estimated this cost at $339 (£233) [39]. The average intervention cost is highly influenced by staff salary costs and the format of the programme, this and the use of marketing in the US may have resulted in the 7% difference. In the NHS marketing cost is not expected as it is assumed that current health care trusts can roll out the programme using existing staff that can be trained.

This study has several limitations. The data on fall occurrence was based on self report and subject therefore to errors of recall, and so our data may underestimate the occurrence of falls in this group. Efforts made to reduce the likelihood of underreporting include the provision of prepaid preaddressed daily calendar postcards to be returned on a monthly basis with follow up calls for non-responders. The effect of any underreporting, however, would be to underestimate the economic burden of the falls. Falls that were reported using the calendars were verified by telephone calls and followed up to gather more information about the type of fall and any injuries. This information was used to categorise the fall according to severity by using both type of injury (fractures, internal bleeding and sprains) and healthcare service utilisation (e.g., hospital admission, stitches, and physiotherapy). Again, however, the data was based on self report and subject therefore to errors of recall. A randomised controlled trial would exclude these errors and give more control over the study. The healthcare costs for a fall was calculated using maximum information accessible, however it is still based on the assumption that certain services was provided which may not have been the case. Additionally the costs-benefit analysis in favour of implementing the OEP holds strictly to the assumptions used for estimating the average cost of the intervention.

We have performed a sensitivity analysis based on removing the costs that we assume, and are not based on the self-reported data. This involved subtracting 14% (the assumed cost; Fig. 2) from the total sum. This gives a total cost sum of £321,944 and hence a net-benefit of £88,8986. However, as we believe the assumptions that we make are realistic, we prefer the main discussion to focus on the full results. It would be highly unlikely for participants who have had serious falls not to have received treatment especially so if they had an overnight stay in the hospital.

The data in this study suggest that management of RA patient should, because of the cost savings, include a fall prevention programme such as the OEP. Given the higher risk of falls among those who have already experienced a fall, it might be offered in the first instance to those with a fall in the previous year. In this study only the OEP has been used and this has not been compared with other exercise programmes. Further research should include a cost-benefit comparison between OEP and other exercise programmes (as well as estimating the costs and efforts involved in undertaking the OEP in a RA specific population). There are other interventions that can be delivered at home by health professionals to maximise effectiveness and reduce falls. These include, assessments and modifications of environmental hazards [40], home safety advice and referral to doctors for re-assessment of psychotropic drugs [41]. The intervention has demonstrated to reduce falls by 35% and reduce moderate and serious injuries by 40%; this can reduce healthcare service utilisation and in turn reduce healthcare costs [41].

## Conclusion

The implementation of the programme for patients with RA has potentially significant economic benefits and should be considered as part of an overall management strategy for patients with the disease. To further investigate and reinforce the findings of this study a randomised controlled trial should be conducted.

## Abbreviations

NHS: National Health Service
NICE: National Institute of Health and Clinical Excellence
NSR: National schedule of reference
OEP: Otago exercise programme
PRI: Positive return of investment
PSSRU: Personal Social Service Research Unit
RA: Rheumatoid arthritis
ROI: Return of investment
